# State-of-the-Art CNN Optimizer for Brain Tumor Segmentation in Magnetic Resonance Images

**DOI:** 10.3390/brainsci10070427

**Published:** 2020-07-03

**Authors:** Muhammad Yaqub, Jinchao Feng, M. Sultan Zia, Kaleem Arshid, Kebin Jia, Zaka Ur Rehman, Atif Mehmood

**Affiliations:** 1 Faculty of Information Technology, Beijing University of Technology, Beijing 100000, China; myaqubciitswl@gmail.com (M.Y.); a_kaleem@outlook.com (K.A.); kebinj@bjut.edu.cn (K.J.); 2 Department of Computer Science and IT, The University of Lahore, Gujrat Campus, Main GT Road, Adjacent Chenab Bridge Gujrat, Gujranwala, Punjab 52250, Pakistan; sultan.zia@cs.uol.edu.pk (M.S.Z.); rao.zaka@yahoo.com (Z.U.R.); 3 Beijing Key Laboratory of Computational Intelligence and Intelligent System, Beijing University of Technology, Beijing 100000, China; 4 School of Artificial Intelligence, Xidian University, No. 2 South Taibai Road, Xi’an 710071, China; atifedu151@yahoo.com

**Keywords:** brain tumor, optimizer, deep learning, convolutional neural network, gradient descent, segmentation, Adam

## Abstract

Brain tumors have become a leading cause of death around the globe. The main reason for this epidemic is the difficulty conducting a timely diagnosis of the tumor. Fortunately, magnetic resonance images (MRI) are utilized to diagnose tumors in most cases. The performance of a Convolutional Neural Network (CNN) depends on many factors (i.e., weight initialization, optimization, batches and epochs, learning rate, activation function, loss function, and network topology), data quality, and specific combinations of these model attributes. When we deal with a segmentation or classification problem, utilizing a single optimizer is considered weak testing or validity unless the decision of the selection of an optimizer is backed up by a strong argument. Therefore, optimizer selection processes are considered important to validate the usage of a single optimizer in order to attain these decision problems. In this paper, we provides a comprehensive comparative analysis of popular optimizers of CNN to benchmark the segmentation for improvement. In detail, we perform a comparative analysis of 10 different state-of-the-art gradient descent-based optimizers, namely Adaptive Gradient (Adagrad), Adaptive Delta (AdaDelta), Stochastic Gradient Descent (SGD), Adaptive Momentum (Adam), Cyclic Learning Rate (CLR), Adaptive Max Pooling (Adamax), Root Mean Square Propagation (RMS Prop), Nesterov Adaptive Momentum (Nadam), and Nesterov accelerated gradient (NAG) for CNN. The experiments were performed on the BraTS2015 data set. The Adam optimizer had the best accuracy of 99.2% in enhancing the CNN ability in classification and segmentation.

## 1. Introduction

**Figure 1:**
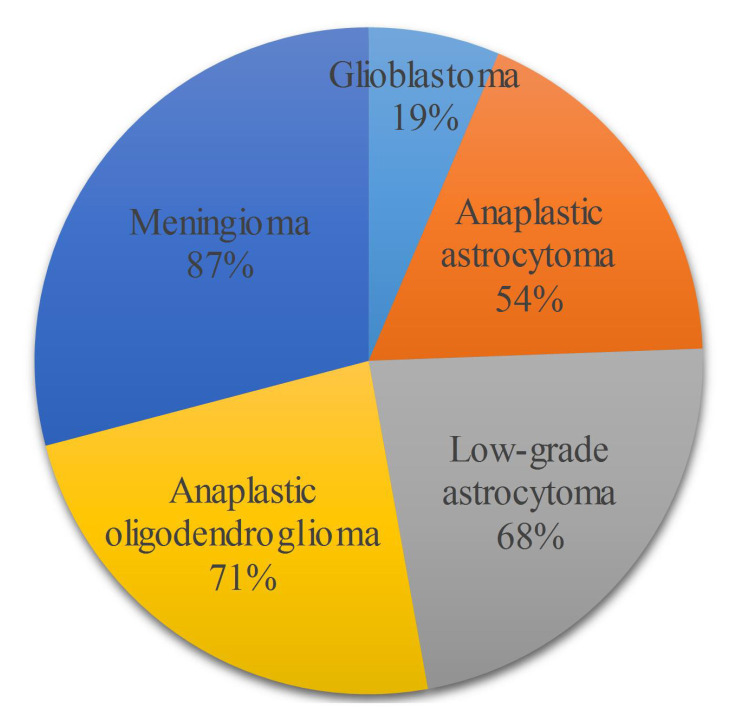
The 5 tumor types with their survival rates for patients aged between 20–44.

A disease is defined as a disorder of function in a living being. If we drill down the definition, it can be defined as a disorder of structure or function in the division of cells in a living organism. If a disorder of unnatural mass is developed in the cerebrum of a brain, we call it a brain tumor. Brain tumors are of different types and can be dangerous at times, and glioma is the most common type of nonpermanent or treatable tumor. Glioma can be classified into two types, namely High-Grade Gliomas (HGG) and Low-Grade Gliomas (LGG). LGG is a slow-spreading tumor, while HGG is a rapidly growing tumor, which explains why HGG is a fatal disease. People who are diagnosed with HGG and who are aged between 20–44 years have a survival rate of 19% with treatment after 14 months of diagnosis, based on a recent survey of the central nervous system (CNS) [1] on a Canadian population from 2009–2013. Figure 1 shows the distribution of survival rates between different types of brain tumors.

**Figure 2:**
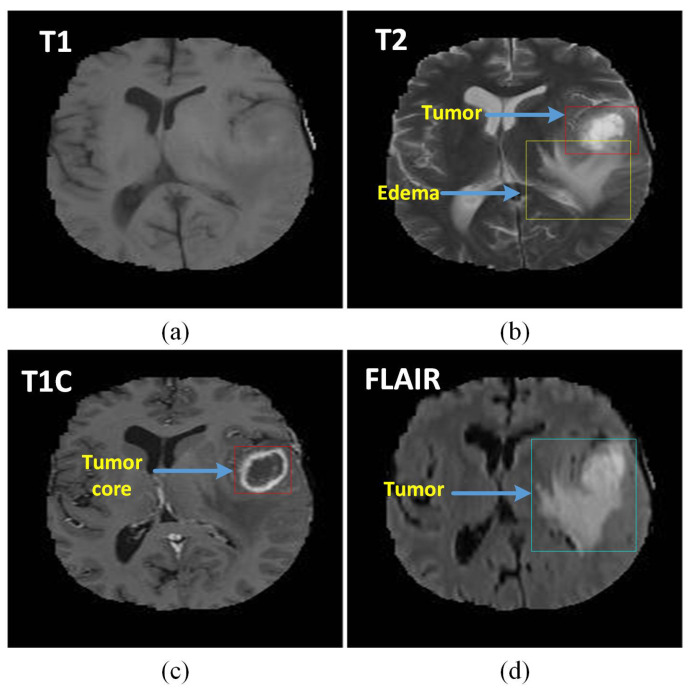
The different tumor types with different shapes in four magnetic resonance images (MRI) sequences: (**a**) T1 MRI sequence, (**b**) T2 MRI sequence with tumor type edema, (**c**) T1C MRI sequence with core tumor, and (**d**) Search Results Web results Fluid attenuation inversion recovery (FLAIR) sequence showing the ground truth of a tumor.

Though there are many medical imaging modalities available to differentiate the characteristics of brain tumors, magnetic resonance images (MRIs) are the most commonly used medical imaging modalities due to its advantage of visual analysis and its flexibility in the domain of computer-aided analysis of medical images. It plays a vital role at many stages of the clinical work flow for population screening; the role of MRI modalities will ramp up in the coming future due to developments in the domain of analysis methods along the lines of cost effectiveness and accuracy. With the help of MRIs, tumors can be differentiated into different grades of gliomas. Among the latest high-tech technologies, MRIs can be considered one of the most advanced techniques used to characterize brain tumors for diagnosis and evaluation. Accurate identification of tumor distance can be considered a critical phase of various neuroimaging studies [2]. The types of MRI modalities are clearly outlined in Figure 2. They [3] focused their experimental analysis on the fully annotated brain tumor segmentation (BraTS) challenge 2013 data set using the well-defined training and testing splits, thereby allowing us to compare directly and quantitatively a wide variety of other methods. Deep learning (DL) and Convolutional Neural Networks (CNN) stood at the center of all these developments in brain MRI image analysis and computer interventions and proved their adoption to be a successful execution to drive for continuous improvements.

Convolutional networks were inspired by biological processes [4,5] in that the connectivity pattern between neurons resembled the organization of the animal visual cortex. Initially, Artifical Neural Network (ANN) was used to study the data from digital images, but in order to do so, the domain experts or the researches have to manually decide and extract features from the digital images and to feed it to the ANN. CNN came to the rescue in eliminating the cumbersome manual work of deciding the features. CNN is one of the most remarkable forms of ANN that is inspired by natural visual recognition phenomenon [6]. There are innumerable applications of CNN in the field of image classification and pattern recognition [7]. The architecture of CNN was introduced in the late 80 s [8]. After the introduction of CNN, it was improved by LeCun in the late 90s [9], but the introduction of the ConvNet architecture [10] in the 21st century has taken CNNs to a different level, with an error rate of 15.3% as compared to conventional computer vision (CV) techniques [11].

CNN has made huge impacts in the medical imaging domain [12] and many other fields such as computer vision, digital image processing, and artificial intelligence. Due to its multilayered architecture, CNN is the most popular technique employed for image analysis although there are many deep learning algorithms introduced over the past decades [13,14,15]. Similar to ANN, CNN also uses an adaptive approach to learn spatial hierarchies of features through back propagation, but unlike ANN, CNN does not have fully connected neurons for all the layers and it has only the last layer as fully connected layer. CNN consists of multiple building blocks, such as convolution layers, pooling layers, and fully connected layers [16]. The convolution layer is responsible for feature extraction, which makes it special compared to ANN; this layer is typically responsible for convolution operation and activation function.

## 2. Literature Review

Over the last few years, there has been significant effort from fellow researchers in the development of CNN; some articles focused on studying the characteristics of machine learning algorithms [17], which tried to explain the multilayered network methodology along the lines of back propagation and updating weights. In Table 1 detailed literature review presented with methodology, result and future directions. The working mechanism of CNN and other neural networks along with their usage in the machine learning and deep learning algorithms is also discussed briefly. Their study opened the door towards future research directives leading to image segmentation. It has made it clear that the acquisition of an edge is a necessary part of a good segmentation [18]. Segmentation usually means segmenting the image into a required partition; edge detection is employed by many researchers to find the best regions in the image. Canny and his coworkers were introduced to edge-based segmentation, which utilized the optimal smoothing filter to maintain the edges while performing image segmentation. In a pilot study conducted by [19], in an effort to monitor the performance of a handwriting recognition algorithm, segmentation was employed to handle the spatially sparse components and these segmented parts were utilized as an input image in the input layer of the CNN algorithm. Reference [20] proposed a deep siamese convolution neural network-based approach for classification of Alzheimer’s disease stages and produced promising results in term of classification on brain images, which is able to identify normal control and disease patients. Reference [15] introduced a thresholding technique, which became a popular technique used for segmentation over the years. They suggested the aforementioned limitation by designing a framework for optimizing Bayesian design and the stability of integrated multidisciplinary systems. Their proposed framework was built on the Gibbs estimation process and applies the gradient information policy (KG) for sequential ordering to achieve the largest one-time increase expected in the design process [21]. In this technique, the gray scale and white scale values are filtered or manipulated by selecting a threshold value. Otsus method, k-means clustering, and maximum entropy method are among the most famous threshold techniques. The threshold value acts as a mid-value, and input values above or below the nominated threshold value are finally displayed. Thresholding methods are nowadays used in segmenting computed tomography (CT), Magnetic Resonance (MR), and Ultrasonic and Positron Emission Tomography (PET) images. In a recent study, a blend of RNN and CNN was introduced with the name Convolutional Recurrent Neural Network (CRNN) [14]. The specialty of CRNN is the recurrent layer, which adds the extracted features, and the feed forward layer, which provides the output. An improved output is obtained due to the improved back propagation. In [22], a 3 CNN layer-based deep learning method was proposed to classify different brain tumour types and grades. Deep-CNN-based transfer learning and fine-tuning was used to segment brain tumors [23]. A recent study clearly outlined the components of CNN (layers, ReLU, dropout, response, and pooling) and its working mechanism [24]; this study is a comparison of scale-invariant feature transform (SIFT) and sparse coding for ImageNet LSVRC (Large Scale Visual Recognition Challenge) held in 2010. A data set of over one million images was utilized with over 1000 categories for training and 50,000 images for testing their system, and they adopted a methodology which enhanced the error rate approximately by 2%. Furthermore, when researchers were struggling with discriminative classifiers by separating the hyper planes, supervised learning-based Support Vector Machine (SVM) came to the rescue. A recent study applied Least Square Support Vector Machine (LS-SVM) to separate the White Matter (WM) and Grey Matter (GM) regions [25] and used a brain atlas for their analysis by performing manual intervention. Later on, scientists started to introduce the multilayered combined multidimensional methodology [26]. The winner neuron was taken as an input after maxpooling the function and took part in further training and segmentation for deep learning. This 2D methodology somehow improved the algorithm performance.

## 3. Optimization Algorithms

Most of the neural network-based techniques including CNN utilize gradient descent to lower the error rate for the training process and for reforming the internal parameters. Gradient descent is a first-order optimization algorithm, and its derivatives provides direction and increasing or decreasing error function. Information guides the error function, altering it downward to the local minimum [29]. The orthodox batch gradient descent technique computes a gradient of the whole training data, which makes its process computationally slow. To overcome this problem, some algorithms were developed as follows.

### 3.1. Adaptive Momentum (Adam)

The Adaptive Momentum (Adam) technique estimates the adaptive learning rate for all parameters involved in the training of gradients. It is a computationally efficient and very simple technique that includes first-order gradients with a small memory requirement for stochastic optimization. The proposed technique is utilized in the case of machine learning issues with high-dimensional parameter spaces and huge data sets that calculate learning rates individually for various parameters from approximations that include first- and 2nd-order moments [14]. The mathematically notation for Adam are as follows:

(1) $$x_{t}=\delta_{1}*x_{t-1}-\left(1-\delta_{1}\right)*g_{t}$$

(2) $$y_{t}=\delta_{2}*y_{t-1}-\left(1-\delta_{2}\right)*g_{t}^{2}$$

(3) $$\Delta \omega_{t}=-\eta \frac{x_{t}}{\sqrt{y_{t}+\epsilon }}*g_{t}$$

(4) $$\omega_{t+1}=\omega_{t}+\Delta \omega_{t}$$

- η: Initial learning rate
- $g_{t}$: Gradient at time *t* along $\omega^{j}$
- $x_{t}$: Exponential average of gradient along $\omega_{j}$
- $y_{t}$: Exponential average of squares of gradient along $\omega_{j}$
- $\delta_{1},\delta_{2}$: Hyperparameters

Adam reduces the computational cost, requires less memory for implementation, and is invariant to diagonal rescaling of the gradients. This takes care of the issues such as but not limited to huge data sets, hyperparameters, noisy data, inadequate gradients, and nonstationary problems that required small tuning. Adam configuration parameters are alpha α: this is a learning rate or step size, most presumably picking large esteem (e.g., 0.3) in light of the actuality that it achieves quick learning instead of a smaller esteem and that the outcomes back adapting are perfect during training.

### 3.2. Stochastic Gradient Descent (SGD)

Stochastic Gradient Descent (SGD) and variants of SGD are commonly used for deep learning. These algorithms have well-defined steps that produce outputs by taking inputs to produce exact results [40,41]. According to cost work and target work, the best algorithms are determined by strategic relapse, linear regression, and neural networks. The main purpose of SGD is to limit the cost work. If there is a huge preparing set, gradient descent is a computationally exceptionally sumptuous system itself. SGD-based algorithms allow users to customize the measures of the algorithms for huge data sets. We performed linear regression utilizing gradient descent, and the equation was as follows:

(5) $$W=\omega -\eta \nabla Q_{i}\left(\omega \right)$$

where $Q_{i}\left(\omega \right)$ is the estimated data, $-Q_{i}$ being the current data under observation. Generally, *Q* is an error function; subsequently, by tracking the gradient direction in the space of values of (ω), we move in the direction of (ω) that reduces the error. SGD computes the best (ω) by minimizing *Q* simultaneously. More importantly, with either perception segmentation or linear regression, (ω) requires the weight parameters of the model and Q(ω) is a the error for the model. Regular gradient descent is composed as follows:

(6) W←η∇Q(ω)

where the error objective is (with its gradient)

(7) Q(ω)=ln∑iQi(ω)⇒∇Q(ω)=ln∑i∇Qi(ω)

### 3.3. Momentum

Stochastic gradient descent is a popular optimization technique, but the run time is comparatively high when training the model. Momentum is intended for quick learning, particularly in the face of high curvatures, small but noisy gradients, or steady gradients. The neighborhood minima can be obtained by the utilization of momentum by the quantity of motion of a moving body with respect to its mass and velocity [42]. This is the add-on of the backpropagation technique that updates the weights by decreasing the error rate from the backward direction. A change of direction in the gradient will change the momentum accordingly. Momentum comes in handy, especially when the network is not well defined. Various directions will cause the development of long tight valleys. In these conditions, the inaccurate surface has a comprehensively unique ebb and flow along a gradient descent that does not point towards the base as most point to the surface and the continuous step size of the GD can vacillate from one side to the next. It progresses very slowly to the minima. The expansion of momentum accelerates the intermingling at least by damping these motions. The weight *w*, momentum *m*, and given time (*t*) become:

(8) $$\Delta \omega_{i,j}=\mu \delta_{i}y_{i}+m\Delta \omega_{i,j}\left(t-1\right)$$

This equation shows that the overall parameters are essentially dictated by experimentations where 0<m<1. Weight refresh presents as one, and momentum adds the fraction *m* that increases step size towards the minimum when the gradient technique suggests a similar solution. The overall learning rate can be reduced when utilizing a great deal of momentum (*m* near 1) by surging past the base with excellent stages, and it is joined by an excessive learning rate with momentum. Momentum provides an updated rule which is inspired by the physical perspective of optimization. Imagine a ball in the mountainous area trying to reach the deepest valley, it passes through slight hills when the slope is very high and the ball gains a lot of momentum. The speed of the ball depends on the momentum of the ball, and momentum provides a boost to speed up learning that changes very little to SGD and velocity to make the updates that store velocity for the parameters. The adapted function for SGD uses the momentum updated rule. However, while momentum is very high, the goal is very close which does not know how to slow down the speed. At the beginning, the oscillate minima do not reach the goal. GD has extra cure surfaces in one direction but not in the other direction. It also reduces the oscillation. For updating the weights, it takes the gradient of the current and previous time steps which move faster towards convergence. Convergence is faster when we apply the momentum optimizer to surfaces with curves.

(9) $$v_{t}=\gamma v_{t-1}+\eta \nabla J\left(\theta ;x,y\right)$$

(10) $$\theta =\theta -v_{t}$$

### 3.4. Adaptive Gradient (Adagrad)

Adagrad adjusts the learning rate according to the parameters, performing bigger updates for inconsistent parameters and smaller updates for successive parameters [43]. The update for each parameter $\theta_{i}$ in each iteration *t* is as follows:

(11) $$\theta_{t,i}=\theta_{t-1,i}-\frac{\eta }{\sqrt{G_{t-1,ii}+\epsilon }}\cdot g_{t-1,i}$$

where $g_{t-1,i}$ is the gradient to the parameter of the target function $\theta_{i}$ at iteration t−1 and ϵ is a smoothing term which dodges division by zero:

(12) $$g_{t-1,i}=\nabla_{\theta }J\left(\theta_{i}\right)$$

where $g_{t-1,ii}\epsilon R_{d\times }$ is a diagonal matrix and element *i* is the sum of the squares of the gradients to $\theta_{i}$ to iteration t−1. By means of an element-wise matrix– vector multiplication ⊙between $G_{t-1}$ and $g_{t-1}$, the vectorization of

(13) $$\theta_{t}=\theta_{t-1}-\frac{\eta }{\sqrt{G_{t-1,ii}+\epsilon }}\cdot g_{t-1,i}$$

Adagrad takes out the requirement to manually tune the learning rate; however, its gathering of the squared gradients in the denominator causes the learning rate to shrivel and to moderate intermingling speed.

### 3.5. Adaptive Delta (AdaDelta)

AdaDelta is an extension of Adagrad which clears the rotting learning rate issue but, unlike Ada Grad, does not collect past squared gradient. It restricts the window of gathered previous gradient [44]. The AdaDelta technique strongly adjusts weights after using the first-order time only and takes minimum computational costs compared to previous techniques. In this technique, there is no manual tuning or learning. Moreover, it is robust to raucous gradient information, data modalities, hyperparameters, and model design decisions. It improves the steepest descent direction stated by a negative gradient.

(14) $$\nabla x_{t}=-\eta g_{t}$$

where $g_{t}$ is the gradient at the *i*th iteration $\frac{\delta f(x_{t}}{\delta f(x_{t}})$ and η is a learning rate which controls how vast the stage is toward the negative gradient. Picking a learning rate and exhibiting another unique learning rate evaluated per measurement by utilizing the first order, it utilizes a modest quantity of calculation per iteration in gradient descent, which is a downside to AdaDelta. Some of their discovered hyper parameters are not up to the best degree to adjust outcomes. Inspired from Adagrad, the two primary disadvantages are

- the incessant rot of learning rates for the training time and
- the requirement for automatically chosen comprehensive learning rates.

Notwithstanding across the board assortment of input data types, nonlinearity, total hidden units, number of distributed imitations, and the hyperparameters that do not need to be balanced are some concrete reasons exhibiting that AdaDelta has strong learning that can be useful in grouped assortment of conditions and that there is no requirements for the physical setting of a learning rate.

### 3.6. Adaptive Max Pooling (Adamax)

Adamax is inspired from Adam; the changes are made on how the infinity norm (ut) is used. It was demonstrated that the $v_{t}$ value in Adam with 1 will merge to a progressively stable value [45].

(15) $$u_{t}=\beta_{2}^{\infty }\cdot v_{t-1}+\left(\beta_{2}^{\infty }\right)\cdot |g_{t}{|}^{\infty }=max(\beta_{2}\cdot v_{t-1},|g_{t}\left|\right)$$

### 3.7. Nesterov Adaptive Momentum (Nadam)

Reference [5] presented a variant of the momentum algorithm inspired by Nesterovs accelerated gradient method [46].

(16) $$v\;\leftarrow \;\alpha v-\epsilon \nabla_{\theta }\left[\frac{1}{m}\sum_{i=1}^{m}L(f\left(x^{\left(i\right)};\theta +\alpha v\right),y^{\left(i\right)}\right)]$$

(17) θ←θ+v

where the parameters α and ϵ play similar roles as in the standard momentum method. The difference between Nesterov momentum and standard momentum is evaluation of the gradient. With Nesterov momentum, the gradient is evaluated after the existing velocity is applied. Thus, one can interpret Nesterov momentum as an attempt to add a correction factor to the standard method of momentum.

### 3.8. Root Mean Square Propagation (RMSProp)

Root Mean Square Propagation (RMSProp) was invented by Geoffrey Hinton. It is similar to the gradient descent algorithm with momentum. RMSProp tries to resolve Adagrad’s radically diminishing learning rates by using a moving average of the squared gradient, which utilizes the magnitude of recent gradient descents for normalization of the gradient. Therefore, with the increase of the learning rate, the algorithm used would move in a horizontal direction with larger steps converging faster.

(18) $$E{\left[g^{2}\right]}_{t}=0.9E{\left[g^{2}\right]}_{t+1}+0.1g_{t}^{2}$$

(19) $$\theta_{t+1}=\theta_{t}-\frac{\eta }{\sqrt{\left(1-\gamma \right)g_{t-1}^{2}}+\gamma g_{t}+\epsilon }\cdot g_{t}$$

γ is the decay term that takes a value from 0 to 1. $g_{t}$ is the moving average of squared gradients.

### 3.9. Cyclic Learning Rate (CLR)

Learning rate is a hyperparameter that controls how much you are adjusting the weights of our network with respect to the loss gradient. It is because you are on your way to optimizing a neural network that you have just created with gradient descent. Now, essentially the goal of gradient descent is to find the minima of the loss function that your neural network is trying to optimize.

- CLR provides a technique for setting the global learning rates for training neural systems that take out the the need to perform tons of investigations to locate the best values with no extra computations.
- CLR provides an excellent learning rate range (LR range) for an experiment by introducing the concept of LR range test.

### 3.10. Nesterov Accelerated Gradient (NAG)

Nesterov acceleration optimization is similar to a ball rolling down the peak but knowing exactly when to slow down before the gradient of the hill increases again. We can calculate the gradient not with respect to the present step but with respect to the future step. We estimate the gradient of the gain, and based on the importance, we will update the weights accordingly. When going down the peak where we can look ahead in the future, we can optimize the descent faster, which is the reason it works slightly better than standard momentum.

(20) $$\theta =\theta -v_{t}$$

(21) $$v_{t}=\gamma v_{t-1}+\eta \nabla J\left(\theta -\gamma v_{t-1}\right)$$

Selection of a good starting learning rate is just the first step. Further, we need to gradually decrease the learning rate while training for a robust model. The learning rate becomes constant during the course of training, which might become too large to converge and causes the loss function to change around the local minimum. This approach is used for a higher learning rate to rapidly reach the regions of (local) minima, while in the initial training stage, a smaller learning rate is set, as training progresses, exploring deeper and more thoroughly in the region to evaluate the minimum.

## 4. Data Set and Methodology

The proposed technique has been trained and validated on the BraTS2015 databases [47]. In BraTS2015, there are four MRI sequences available for every patient: FLAIR, T1-weighted (T1), T2-weighted (T2), and T1-weighted (T1c). The training set involves 220 High-Grade Gliomas (HGG) and 54 Low-Grade Gliomas (LGG) in the BraTS2015 challenge data set. We extracted around 268,000 and 360,000 patches to train our proposed CNNs for LGG and HGG, respectively. The proposed CNNs were developed using Tensorflow backend.

**Figure 3:**
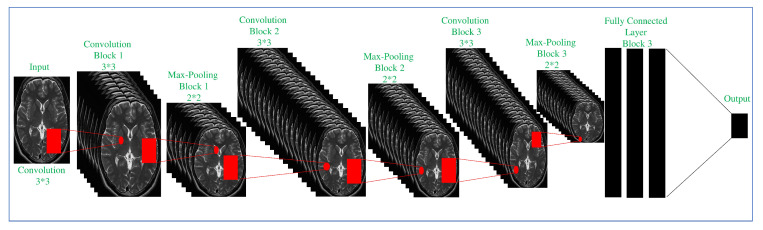
Proposed architecture.

The proposed ConvNet CNN architecture was utilized in the investigation. In order to get enough information on the optimizer’s performance and accuracy, the proposed convNet architecture was trained and validated with different optimizers explained in the above section. We divided the data set in Table 2 for training 80% and validation 20%. The ConvNet architecture was validated using 10 optimizers based on gradient descent (as shown in the above section), 2 options of the data batch size (128), 3 options of the epoch (0,50,…250,300), and 4 options of the learning rate (1e${}^{-1}$, 1e${}^{-2}$ … 1e${}^{-10}$). We used the patches of the different patients in the training and validation processes. Details of the architecture are given in Table 3. The proposed ConvNet architecture consists of 8 convolution layers, 3 max-pooling layers, and 3 fully connected layers. Some activation functions used in the ConvNet architecture include Rectified Linear Unit (ReLU) and Soft Max. The pooling layer is responsible for dimensionality reduction over time; it reduces the spatial size in every step to decrease the number of selected parameters for the subsequent step by operating on each feature map independently. The output from the final pooling or convolution layer follows a one-dimensional (1D) array; from here, the architecture is completely the same as ANN, in which, all the values in the 1D array are fully connected to every output by a temporary weight given to each of them. Each fully connected layer is followed by a nonlinear function; the fully connected layers typically have the same number of output nodes as the number of classes. Design details can be found in Table 3, and the ConvNet architectural is shown in Figure 3.

## 5. Experimental Results and Discussion

We used Tensorflow library for the implementation of our model on Z840 workstation Intel Xeon (R) CPU E5-2630v3 @2.40GHz*32 with 64 GB memory. To validate the effectiveness, the proposed CNN-based approach with extra convolutional layers was used to classify brain tumor disease. We used the Monte Carlo method to check the significance of the classification and segmentation results under optimal parameters. We performed the analysis on the different number of epochs, and we noticed that the average performance results were achieved on 300 epochs. Table 4 shows the hyperparameters of our proposed technique.

Table 5, Table 6, Table 7, Table 8, Table 9 and Table 10 show a summary of the experiment results. The experimental results uses ten gradient descent optimizers with different number of epochs and various learning rates. Table 5, Table 6, Table 7 and Table 8 give the results at epochs 50, 100, 200, and 300. Table 9 and Table 10 provide the results with learning rate 1e${}^{-1}$, 1e${}^{-2}$, … 1e${}^{-10}$.

**Figure 4:**
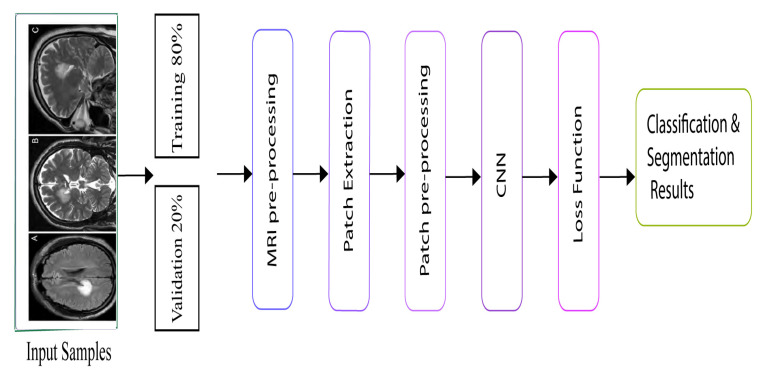
Proposed model flow chart.

**Figure 5:**
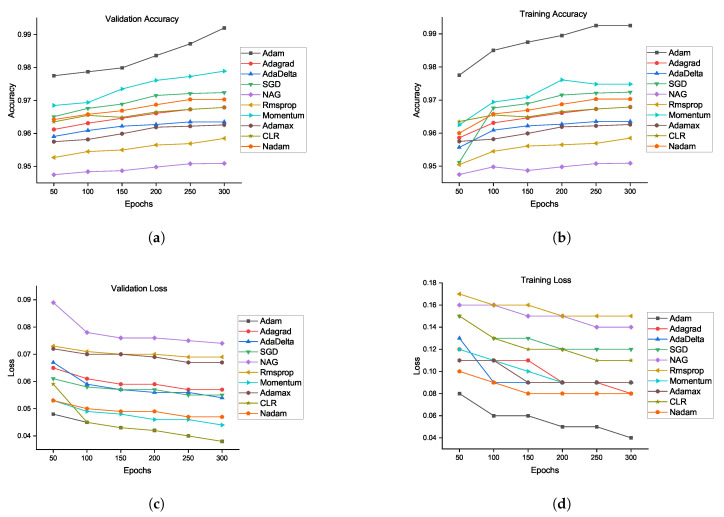
Validation accuracy and loss comparison of all optimizers using our proposed architecture: (**a**) validation accuracy, (**b**) training accuracy, (**c**) validation loss, and (**d**) training loss.

The flowchart of the proposed method is defined in Figure 4. There is likewise no sign of overfitting, implying that the architecture performs well. The smallest error rate was obtained by the Adam optimizer using our proposed CNN architecture. Adam is the most successful optimizer in our all experiments. Other optimizers also performed well, and the performances of SGD and momentum are close to that of Adam. Figure 5 shows the performances of ten optimizers using CNN architecture with various epochs and learning rates. From Figure 5a, we can observe that Adam, AdaDelta, and SGD provide the highest validation rates at 300 epochs. Figure 5b shows that Adam has the highest training accuracy whereas NAG has the lowest training accuracy. Other than Adam, momentum and SGD likewise have the potential to accomplish high training accuracy. Figure 5c shows that Adam provides the smallest validation loss whereas RMSProp, NAG, and Adamax have the biggest validation losses. From Figure 5d, we can see that RMSProp, NAG, and CLR provide the highest trainng losses whereas Adam has the smallest training loss. Henceforth, it could be construed that each optimizer shows diverse execution crosswise over various epochs using CNN architecture. A comparison of our proposed method has been presented in Table 11. Reference [29] experimented on different auto-encoders to demonstrate that NAG has better abilities in terms of reducing the gradient norms, and it also produces iterates which exhibit an increasing trend for the minimum eigenvalue of the Hessian of the loss function at the iterates. Four classes of the CIFAR-10 data set are chosen for the experiments.The proposed EIOM is compared with other optimizers, namely AdaGrad, AdaDelta, RMSProp, Adam, and CLR that produced 97% accuracy [31]. Extensive experiments on four widely used benchmark databases were conducted to verify the effectiveness of the proposed deep convolutional neural network (DCNN) and obtained 97.9% accuracy.

**Figure 6:**
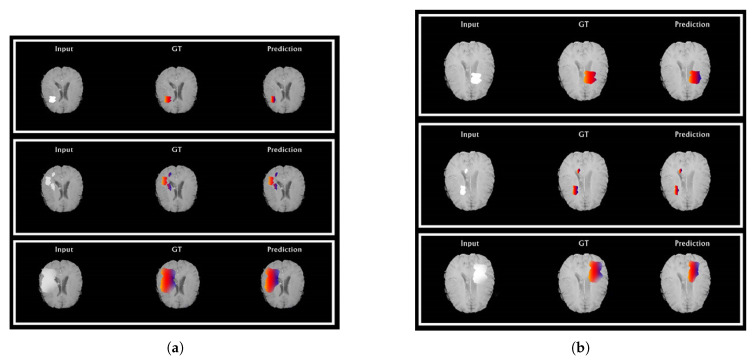
Automatic segmentation results of HGG (**a**) and LGG (**b**) cases. Red: edema, blue: non-enhancing tumor. From left to right: (i) original image, (ii) ground truth, and (iii) demonstration of automatic segmentation results from the proposed method. (**a**) The segmentation results of HGG cases compared to their ground truth and (**b**) the segmentation results of LGG cases compared to their ground truth.

**Figure 7:**
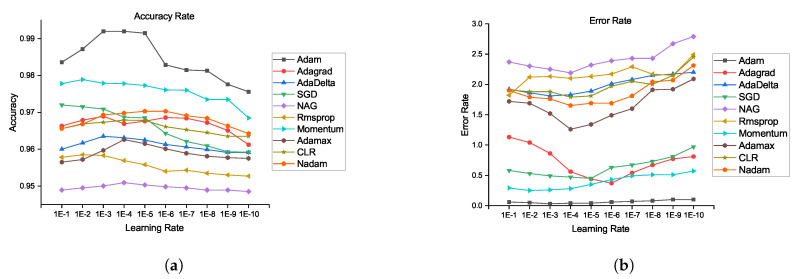
Accuracy rate and error rate comparison of all optimizers using our proposed architecture: (**a**) accuracy rate and (**b**) error rate.

From Figure 6a,b, the segmentations results for the HGG and LGG cases are compared with the delineated ground truth: (a) segmented tumor of a HGG case overlaid on the FLAIR image; (b) segmented tumor of a LGG case overlaid on the Flair image. From Figure 7a,b, further examination of the potential optimizers for CNN architecture is delineated. Some of learning rates were added to portray its conduct against the likelihood of overtraining. As much as 1e${}^{-1}$, 1e${}^{-2}$ … 1e${}^{-10}$ learning rate was experimented to train our proposed CNN with all optimizers. It was demonstrated that all optimizers did not require much learning rate to arrive at the minimum error rate and that all optimizers could converge well overall. In any case, Adam is the most steady one among the ten optimizers. Then again, NAG, RMSProp, and CLR were not successful to do as such. The error rate of SGD and momentum tended to diminish when the learning rate was smaller than 1e${}^{-5}$. Compared to Nadam, Adagrade and AdaDelta have worse performance. Therefore, Adam is the best choice for brain tumor segmentation using our proposed CNN architecture. From the above discussion, it is uncovered that our proposed CNN architecture with various optimization algorithms gives impressive results for brain tumour segmentation using MRIs. From the pack of our experiment data, it is conceivable to explain the behaviour of each optimizer against CNN architecture. Finally, it can be seen that our proposed model achieves state-of-the-art results when comparing it with existing models. We also provide an in-depth analysis of our proposed method and produce 99.20% accuracy.

## 6. Conclusions

This paper is a comparative analysis of different optimization algorithms used in our proposed CNN architecture to measure the performance for brain tumor segmentation. The comparison is made on publicly available an MRI brain image data set, i.e., BraTS2015. Both quantitative and graphical results show that all optimizers perform consistently but that Adam performs much better. Among the 10 optimizers for our architecture, Adam has the smallest error rate and the highest accuracy rate when it reaches the minimum on a particular epoch. The NAG and RMSProp optimizers failed badly. Due to limited resources to run several architectures, AdaDelta and Adamax should be used to provide minimal risk. The performances of the momentum and SGD optimizers were inferior to that of Adam. The adapted pipeline of the CNN optimizer comparison concludes that the performance of Adam is comparable with the latest research. Future work will compare this state-of-the-art optimizer with multiple CNN architectures used for brain tumor segmentation.

## Figures and Tables

**Table 1:** Literature review.

Sr. No.	Methodology	Results	Future Directions
1	Three layered feed forward ANNs and two real world problems are set as a benchmark to access the performance of Group Search Optimizer (GSO) [27].	GSOANN has a far better performance as compared to regular ANN.	**—–**
2	A hybrid model of DSA and DL to help improve the relationship of computer science and bioinformatics [28].	Differential Search Algorithm (DSA) and DL can help produce more xylitol for sugar free gums.	Computational biologists and computer scientist can together produce a hybrid model using deep learning OA.
3	In auto-encoders like VVG-9 and CIFAR-10, they design some experiments to study the properties of RMSProp and Adam against Nesterov’s Accelerated Gradient method [29].	On very high values of β1 = 0.99 Adam outperforms lower training and test losses, whereas with β1 = 0.9, NAG performs better.	Advance theory in getting more better results by getting β1 close to 1.
4	Different optimization algorithms are studied by side CNN architecture [30].	Among 7 optimizers, on the LeNet architecture, Adam provides the smallest MSE whereas SGD and Adagrad failed.	Can build analytical protable image devices
5	Constructed a few illustrative binary classification problems and examined empirical generalization capability of adptive methods agaisnt GD.	Solutions found by adaptive methods generalize worse than GSD.	Adaptive methods should be reconsidered.
6	Energy Index based Optimization Method (EIOM) that automatically adjusts the learning rate in backpropagation [31].	EIOM proves to be the best when compared with state-of-the-art optimzation methods.	—–
7	A non-asymptotic analysis of the convergence of two algorithms: SGD and simple averaging [32].	The analysis suggests that the learning rate is proportional to the inverse of the number of iterations.	Differential and non-differential stochastic
8	Adaptive learning rate and laplacian approach have been proposed for Deep Learning in MLP [33].	Improved classification accuracy	—–
9	Proposed a fundamental approach for anatomical, celluler stuctures, and tissue segmentation using CNN through image patches measuring 13 × 13 voxels [34].	On different data sets, comparing the six commonly used tools (i.e., ROBEX, HWA, BET, BEaST, BSE, and 3dSkullStrip), they achived the highest average specifity.	Can be performed on most advanced tools and used a real time data set to get better result.
10	Used a pretrained CNN model on augmented and orginal data for brain tumor classification [35]	They achieved 90.67 accuracy before and after data augmentation on the proposed methed and compared with most advanced methods	Used light weight CNN to entend their work for fine-grained classification differential stochastic.
11	A CapsNet for brain tumor classification and investigation of the overfitting problem based on CapNet [36].	On 10 epochs, they achieved 86.56% accuracy, with the comparative analysis with CNN learning rate proportional to the inverse of the number of iterations.	In the future, investigations on the effects of more layers on the classification accuracy will be performed.
12	A review on deep learning techniques in the field of medical images classification [37]	They discussed in detail the deep learning approaches and their suitability for medical images. The learning rate is proportional to the inverse of the number of iterations.	Further research is required to apply the techniques to the modalities, where these are not applied.
13	GA-SVM and PSO-SVM method used to classify heart disease [38].	GA and particle swarm optimization (PSO) algorithms combined with SVM achieved a high accuracy.	—–
14	Applied U-NET approach using BraTS2017 data set and prediction of patient survival [39]	89.6% Accuracy achieved with less computational time	—–
15	Two-way path architecture based on CNN for brain tumor segmentation on the BraTS 2013 and 2015 data sets [3]	Input cascaded CNN got a high accuracy with 88.2% on the comparitive analysis with other architechtures.	Further improved the results with increasing architechture layers and data set.

**Table 2:** Data set used for proposed technique: Four MRI modalities are used for both tumor types High-Grade Glioma (HGG) and Low-Grade Glioma (LGG). The modalities are T1-weighted (T1), 2-weighted (T2), T1-weighted (T1c), and FLAIR. The initial two parameters represent the tumor type and number of patients, and the next parameter represents the number of patches extracted for training and validation.

Tumor Type	No. Patients	No. Patches Extracted	
		**Training**	**Testing**
HGG	220	360,000	90,000
LGG	54	268,000	67,000

**Table 3:** Detail of the proposed patch-wise model architecture of Convolutional Neural Network (CNN): In inputs, the first dimension refers to the number of channels and the next two are the size of patch and feature maps, respectively.

Block	No.of Filter	Name (Size)	Stride	Kernel Size
Input		Input Image		-
Convolution block 1	64	Con-1ayer 1 (4 × 33 × 33)	1 × 1	3 × 3
	-	Relu-1ayer		-
	64	Con-1ayer 2 (33 × 33 × 64)		3 × 3
	-	Relu-1ayer		-
	64	Con-1ayer 3 (33 × 33 × 64)		3 × 3
	-	Relu-1ayer		-
Pooling block 1	-	Max-Pooling layer 4 (33 × 33 × 64)	2 × 2	3 × 3
Convolution block 2	128	Con-1ayer 5 (4 × 33 × 33)	1 × 1	3 × 3
	-	Relu-1ayer		-
	128	Con-1ayer 6 (33 × 33 × 64)		3 × 3
	-	Relu-1ayer		-
	128	Con-1ayer 7 (33 × 33 × 128)		3 × 3
		Relu-1ayer		-
Pooling block 2	-	MAX-Pooling layer8 (33 × 33 × 128)	2 × 2	3 × 3
Convolution block 3	128	Con-1ayer 9 (33 × 33 × 128)	1 × 1	3 × 3
	-	Relu-1ayer		-
	128	Con-1ayer 10 (33 × 33 × 128)		3 × 3
	-	Relu-1ayer		-
Pooling block 3	-	MAX-Pooling layer 11 (33 × 33 × 128)	2 × 2	3 × 3
Fully Connected block	-	FC-1ayer 12 32768	-	-
	-	FC-1ayer 13 256		-
	-	FC-1ayer 14 256		-
	-	Softmax-1ayer		-

**Table 4:** Hyperparameters for our proposed technique.

Stage	Hyperparameter	Value
	Bias	0.1
	Weights	Xavier
**ReLU**	α	0.333
**Dropout**	HGG	0.1
	LGG	0.5
**Training**	Epochs-HGG	50–300
	Epochs-LGG	50–300
	Intial ϵ	0.03
	Final ϵ	0.0003
	Batch Size	128
**Post processing**	Tvol-HGG	10,000
	Tvol-HGG	3000

**Table 5:** The training accuracy of ten optimizers on a proposed patch-wise model architecture of CNN.

Epoch →	50	100	150	200	250	300
Optimizers ↓						
Adam	0.97	0.98	0.98	0.98	0.99	0.99
Adagrad	0.95	0.96	0.96	0.96	0.96	0.96
AdaDelta	0.95	0.96	0.96	0.96	0.96	0.96
SGD	0.95	0.967	0.968	0.97	0.97	0.97
NAG	0.94	0.94	0.94	0.94	0.95	0.95
Rmsprop	0.95	0.95	0.95	0.95	0.95	0.95
Momentum	0.96	0.96	0.97	0.97	0.974	0.97
Adamax	0.95	0.95	0.95	0.96	0.96	0.96
CLR	0.96	0.96	0.96	0.96	0.96	0.96
Nadam	0.96	0.96	0.96	0.96	0.97	0.97

**Table 6:** Validation accuracy of ten optimizers on a proposed patch-wise model architecture of CNN.

Epoch →	50	100	150	200	250	300
Optimizers ↓						
Adam	0.97	0.97	0.97	0.98	0.98	0.99
Adagrad	0.96	0.96	0.96	0.96	0.96	0.96
AdaDelta	0.95	0.96	0.96	0.96	0.96	0.96
SGD	0.96	0.96	0.96	0.97	0.97	0.97
NAG	0.94	0.94	0.94	0.94	0.95	0.95
Rmsprop	0.95	0.95	0.95	0.95	0.95	0.95
Momentum	0.96	0.96	0.97	0.97	0.97	0.97
Adamax	0.95	0.95	0.95	0.96	0.96	0.96
CLR	0.96	0.96	0.96	0.96	0.96	0.96
Nadam	0.96	0.96	0.96	0.96	0.97	0.97

**Table 7:** Validation loss of ten optimizers on a proposed patch-wise model architecture of CNN.

Epoch →	50	100	150	300	250	300
Optimizers ↓						
Adam	0.05	0.05	0.04	0.04	0.04	0.04
Adagrad	0.07	0.06	0.06	0.06	0.06	0.06
AdaDelta	0.07	0.06	0.06	0.06	0.06	0.05
SGD	0.06	0.06	0.06	0.06	0.06	0.06
NAG	0.09	0.08	0.08	0.08	0.08	0.07
Rmsprop	0.07	0.07	0.07	0.07	0.07	0.07
Momentum	0.05	0.05	0.05	0.05	0.05	0.04
Adamax	0.07	0.07	0.07	0.07	0.07	0.07
CLR	0.06	0.05	0.04	0.04	0.04	0.04
Nadam	0.05	0.05	0.05	0.05	0.05	0.05

**Table 8:** Training loss of ten optimizers on a proposed patch-wise model architecture of CNN.

Epoch →	50	100	150	300	250	300
Optimizers ↓						
Adam	0.08	0.06	0.06	0.05	0.05	0.04
Adagrad	0.12	0.11	0.11	0.09	0.09	0.08
AdaDelta	0.13	0.09	0.09	0.09	0.09	0.09
SGD	0.15	0.13	0.13	0.12	0.12	0.12
NAG	0.16	0.16	0.15	0.15	0.14	0.14
Rmsprop	0.17	0.16	0.16	0.15	0.15	0.15
Momentum	0.12	0.11	0.10	0.09	0.09	0.09
Adamax	0.11	0.11	0.09	0.09	0.09	0.09
CLR	0.15	0.13	0.12	0.12	0.11	0.11
Nadam	0.10	0.09	0.08	0.08	0.08	0.08

**Table 9:** Accuracy rate of ten optimizers with various learning rates on a proposed patch-wise model architecture of CNN.

Learning Rate →	1e${}^{-1}$	1e${}^{-2}$	1e${}^{-3}$	1e${}^{-4}$	1e${}^{-5}$	1e${}^{-6}$	1e${}^{-7}$	1e${}^{-8}$	1e${}^{-9}$	1e${}^{-10}$
Optimizers ↓										
Adam	0.99	0.99	0.99	0.98	0.98	0.98	0.98	0.98	0.98	0.98
Adagrad	0.97	0.97	0.97	0.97	0.97	0.97	0.96	0.97	0.97	0.96
AdaDelta	0.96	0.96	0.96	0.96	0.96	0.96	0.96	0.96	0.96	0.96
SGD	0.97	0.97	0.97	0.96	0.96	0.96	0.96	0.96	0.96	0.96
NAG	0.95	0.95	0.95	0.95	0.95	0.95	0.95	0.95	0.95	0.95
Rmsprop	0.96	0.96	0.96	0.95	0.95	0.95	0.95	0.95	0.95	0.95
Momentum	0.98	0.98	0.98	0.98	0.98	0.97	0.97	0.98	0.97	0.97
Adamax	0.96	0.96	0.96	0.96	0.96	0.96	0.96	0.96	0.96	0.96
CLR	0.97	0.97	0.97	0.97	0.97	0.96	0.96	0.97	0.96	0.96
Nadam	0.97	0.97	0.97	0.97	0.97	0.97	0.96	0.969	0.97	0.96

**Table 10:** Error rate of ten optimizers with various learning rates on a proposed patch-wise model architecture of CNN.

Learning Rate →	1e${}^{-1}$	1e${}^{-2}$	1e${}^{-3}$	1e${}^{-4}$	1e${}^{-5}$	1e${}^{-6}$	1e${}^{-7}$	1e${}^{-8}$	1e${}^{-9}$	1e${}^{-10}$
Optimizers ↓										
Adam	0.05	0.03	0.04	0.06	0.07	0.1	0.1	0.07	0.1	0.1
Adagrad	1.04	0.86	0.56	0.37	0.54	0.77	0.81	0.54	0.77	0.81
AdaDelta	1.86	1.81	1.83	2.01	2.08	2.17	2.2	2.08	2.17	2.2
SGD	0.53	0.49	0.47	0.63	0.67	0.81	0.97	0.67	0.81	0.97
NAG	2.3	2.25	2.19	2.39	2.43	2.67	2.79	2.43	2.67	2.79
Rmsprop	2.12	2.13	2.1	2.17	2.29	2.15	2.49	2.29	2.15	2.49
Momentum	0.25	0.26	0.28	0.43	0.49	0.51	0.57	0.49	0.51	0.57
Adamax	1.69	1.52	1.26	1.49	1.6	1.92	2.09	1.6	1.92	2.09
CLR	1.88	1.88	1.79	1.97	2.05	2.15	2.45	2.05	2.15	2.45
Nadam	1.79	1.76	1.65	1.69	1.81	2.07	2.31	1.81	2.07	2.31

**Table 11:** Comparison of accuracy values with state-of-the-art techniques.

Paper	Method	Data Set	Accuracy
[43]	CIFAR ConvNet	Mnist Dataset	90.00%
[48]	DCNN(AlexNet)	ILSVRC2012	97.90%
[29]	VGG-9	CIFAR-10	99.00%
[31]	2D-CNN	CIFAR-10	97.00%
**Proposed Method**	ConvNet based	BraTS2015	99.20%

## Abbreviations

The following abbreviations are used in this manuscript:

DLA	Deep learning algorithms
CNN	Convolutional Neural Network
MRI	Magnetic Resonance Images
Adagrad	Adaptive Gradient
AdaDelta	Adaptive Delta
SGD	Stochastic Gradient14Descent
Adam	Adaptive Momentum
CLR	Cyclic Learning Rate
Adamax	Adaptive Max Pooling15
RMS Prop	Root Mean Square Propagation
NADAM	Nesterov Adaptive Momentum
NAG	Nesterov accelerated gradient
HGG	High-Grade Gliomas
LGG	Low-Grade Gliomas
CNS	Central nervous system
ANN	Artificial Neural Network
GT	Ground Truth
CV	Computer Vision
PSO	Particle Swarm Optimization
FLAIR	Fluid Attenuation Inversion Recovery
SIFT	Scale-Invariant Feature Transform
DCNN	Deep Convolutional Neural Network
